# Dual inhibition of EGFR and mTOR pathways in small cell lung cancer

**DOI:** 10.1038/sj.bjc.6605761

**Published:** 2010-08-03

**Authors:** K Schmid, Z Bago-Horvath, W Berger, A Haitel, D Cejka, J Werzowa, M Filipits, B Herberger, H Hayden, W Sieghart

**Affiliations:** 1 Clinical Institute of Pathology, Medical University of Vienna, Vienna, Austria; 2 Institute of Cancer Research, Department of Medicine I, Medical University of Vienna, Vienna, Austria; 3 Department of Internal Medicine III, Medical University of Vienna, Waehringer Guertel 18-20, A-1090, Vienna, Austria

**Keywords:** small cell lung cancer, EGFR, mTOR, erlotinib, RAD001, targeted therapy

## Abstract

**Background:**

In this report we investigated the combination of epidermal growth factor receptor (EGFR) and mammalian target of rapamycin (mTOR) pathway inhibition as a possible new therapeutic strategy for small cell lung cancer (SCLC).

**Methods:**

EGFR, p-AKT, p-ERK, p-mTOR and p-p70s6K protein expressions were studied by immunohistochemistry in 107 small cell lung carcinomas and correlated with clinicopathological parameters. Cells of SCLC were treated with erlotinib±RAD001 and analysed for cell viability, proliferation, autophagy, and pathway regulation.

**Results:**

Epidermal growth factor receptor, p-AKT, p-ERK, p-mTOR, and p-p70s6K were expressed in 37, 24, 13, 55 and 91% of the tumour specimens of all SCLC patients, respectively, and were not associated with disease-free or overall survival. The expression of EGFR was lower in neoadjuvant-treated patients (*P*=0.038); mTOR pathway activation was higher in the early stages of disease (*P*=0.048). Coexpression of EGFR/p-mTOR/p-p70s6K was observed in 28% of all patients . EGFR immunoreactivity was associated with p-ERK and p-mTOR expression (*P*=0.02 and *P*=0.0001); p-mTOR immunoreactivity was associated with p-p70s6K expression (*P*=0.001). Tumour cells comprised a functional EGFR, no activating mutations in exons 18–21, and resistance to RAD001 monotherapy. We found synergistic effects of erlotinib and RAD001 combination therapy on the molecular level, cell viability, proliferation and autophagy.

**Conclusions:**

The combined inhibition of EGFR/mTOR pathways could be a promising approach to treat SCLC.

The options for successful treatment of small cell lung cancer (SCLC) are still very poor. From the time of diagnosis, the median survival rates for SCLCs are 15–20 (limited disease SCLC) and 8–13 months (extended disease SCLC), respectively (Puglisi *et al*, 2010); therefore, new therapeutic strategies are urgently needed.

The epidermal growth factor receptor (EGFR) pathway is a well-known molecular target in several human tumours. Small molecules inhibiting EGFR such as erlotinib or gefitinib as well as anti-EGFR antibodies such as cetuximab were successfully tested in non-SCLC, head and neck, pancreatic and colon cancer (Ciardiello and Tortora, 2008).

Until now, the EGFR pathway has not been studied extensively in SCLC. There are little and controversial data about the presence of EGFR in SCLC tissue (Fischer *et al*, 2007). It was shown that treatment of SCLC cell lines with a monoclonal EGFR antibody reduced invasiveness of tumour cells *in vitro* (Damstrup *et al*, 1998). The tyrosin kinase inhibitor gefitinib, directed against the tyrosin kinase domain of the EGFR, showed single-agent activity against SCLC cells (Tanno *et al*, 2004), and reverted resistance to the chemotherapeutic topotecan *in vitro* (Nakamura *et al*, 2005), but failed to show clinical benefit in a recent phase II clinical trial in patients with SCLC (Moore *et al*, 2006). These data suggest that targeting a single pathway in SCLC may not be efficient enough for successful treatment of this deadly disease.

The mammalian target of rapamycin (mTOR) was intensively studied in a multitude of human tumour entities over the past couple of years. Different mitogens activate AKT, which controls mTOR activation by phosphorylation of TSC2, a component of tuberous sclerosis complexes 1 and 2. Activated mTOR phosphorylates 4EBP-1 and p-p70s6K, which leads to protein translation and tumour growth (Bjornsti and Houghton, 2004). AKT can also be activated by the EGFR (Ono and Kuwano, 2006), which represents a possible molecular link between the mTOR and the EGFR pathways.

The mTOR inhibitors CCI-779 and everolimus (RAD001) have already been approved for treatment of advanced renal cancer (Hudes *et al*, 2007; Motzer *et al*, 2008); together with other mTOR inhibitors, they are under clinical investigation for several other cancer indications. Although there is evidence that the mTOR pathway is active in SCLC cell lines (Fischer *et al*, 2007), mTOR pathway expression in SCLC tissue has not been investigated until now. The mTOR pathway may be involved in mechanisms of SCLC cells to escape cell death after treatment with DNA-damaging agents, as the mTOR inhibitor CCI-779 restored sensitivity of SCLC cells to cisplatin treatment (Belyanskaya *et al*, 2005; Wu *et al*, 2005). However, similar to gefitinib therapy, CCI-779 and RAD001 monotherapy achieved only little benefit for SCLC in recent phase II clinical trials (Pandya *et al*, 2005; Owonikoko *et al*, 2008).

Given the molecular connection of the EGFR and mTOR pathways, we hypothesised that dual inhibition of both pathways may be a suitable new strategy to treat SCLC. Thus, we investigated single and coexpression of both pathways in 107 SCLC tissue samples, their correlation with clinical–pathological parameters, and analysed efficacy of anti-EGFR therapy plus mTOR inhibition in SCLC cell lines.

## Materials and methods

### Patients and tissue samples

The study comprised 107 patients (69 males and 38 females), median age 62 years (range 35–92 years), who underwent surgery for SCLC at the Department of Cardio-Thoracic Surgery, Medical University of Vienna, Austria. After surgery, tissue samples were fixed in 7.5% buffered formalin and embedded in paraffin for routine diagnostics. Neuroendocrine tumour differentiation was confirmed by immunohistochemical staining for neuroendocrine markers (chromogranin A and/or synaptophysin). Tumours were staged according to the International Union Against Cancer (UICC) 2004 issue of the TNM system and revealed pT1 in 36 (34%), pT2 in 55 (51%), pT3 in 4 (4%) and pT4 in 12 (11%) cases; lymph node metastases were present in 57 (53%) cases (pN1, pN2 and pN3 in 30, 21 and 2%, respectively). TNM stages corresponded to UICC stages IA, IB, IIA, IIB, IIIA and IIIB in 22 (21%), 23 (21%), 9 (8%), 18 (17%), 22 (21%) and 13 (12%) cases, respectively. The clinical follow-ups of the patients were retrospectively collected from the archives of the Department of Cardio-Thoracic Surgery, Medical University of Vienna, and respective hospitals performing their follow-ups; median-disease free and overall survival were 11.5 and 20.5 months, respectively (both range 0–179.9 months). In all, 20 (19%) patients received preoperative chemotherapy (cisplatin and etoposid). Adjuvant chemotherapy was known for 50 (47%) patients (cisplatin and etoposid, cisplatin and adriamycin or cyclophosphamid and oncovin; in some cases topotecan was given in second line). In all, 56 (52%) patient suffered tumour recurrence and 48 (45%) died of tumour progression during the observation period.

### Immunohistochemistry

Immunohistochemistry was performed on 4 *μ*m paraffin sections from one representative tissue block per patient stored at the archives of the Department of Pathology, Medical University of Vienna, using antibodies against EGFR (DAKO, Glostrup, Denmark) phospho-mTOR, phospho-p70s6K, phospho-AKT and phospho-ERK (all from Cell Signaling Technology, Danvers, MA, USA).

Epidermal growth factor receptor kit immunostaining was performed according to the manufacturer's instructions. Pretreatment was protease (Sigma, Steinheim, Germany) for p-AKT immunostaining, microwave (5 × 5 min, 900 W) for p-ERK immunostaining, and autoclave (20 min, 1 bar) for p-mTOR and p-p70s6K immunostaining. After blocking, samples were incubated with primary antibodies for 1 h at room temperature. Dilutions for anti-p-AKT, anti-p-ERK, anti-p-mTOR and anti-p-p70s6K were 1 : 25, 1 : 100, 1 : 100 and 1 : 400, respectively. After applying a biotinylated secondary antibody and tertiary reagent (Vector-Laboratories, Bulingam, CA, USA) the antibody binding was visualised by diaminobenzidine (Serva, Heidelberg, Germany) and H_2_O_2_. Colon carcinoma tissue served as positive control. Negative controls were treated with isotype IgG control antibodies. Immunoreactive tumour cells were counted in 10 high-power fields ( × 400) by KS and ZB independently. The scoring system integrated intensity and extent of immunostaining: the number of positive tumour cells was scored 0 (<10%), 1 (10–24%), 2 (25–49%), 3 (50–79%) and 4 (80–100%). Intensity of staining was scored 0 (negative), 1 (weak) or 2 (strong). The results of the extent and intensity of staining of tumour cells were summarised to assess the final score.

### Chemicals

RAD001 (everolimus) was provided by The Novartis Institutes for BioMedical Research Basel, Oncology, Switzerland. Erlotinib was dissolved in DMSO and provided by Professor Thomas Grunt (Department of Medicine I, Institute of Cancer Research, Medical University of Vienna, Austria). Controls were treated with appropriate concentrations of DMSO.

### Cell culture

Two SCLC cell lines were used: GLC-4, donated by Dr EG deVries, Groningen, The Netherlands (Zijlstra *et al*, 1987), and VL-68 (Berger *et al*, 2001). Cells were grown in RPMI-1640 medium supplemented with 10% heat-inactivated fetal calf serum and with 1% penicillin–streptomycin in a humidified atmosphere containing 5% CO_2_ (all from Gibco Life Technologies, Paisley, UK). Cell counts were determined using a CC-108 microcellcounter (Sysmex, Kobe, Japan). Cells growing in logarithmic phases of growth were used for all the studies described below.

### MTT assay

Cells (2 × 10^5^ cells per well) in the logarithmic phase of growth were plated in 0.5 ml complete medium in 48-well plates and allowed to attach overnight. The next day, RAD001, erlotinib or a combination of both were added at concentrations as indicated. The final concentration of DMSO never exceeded 0.5%. Cells were incubated for 72 h at 37 °C in a humidified atmosphere containing 5% CO_2_. Afterwards, medium was replaced by 100 *μ*l Opti-MEM medium containing 1 mg ml^–1^ Thiazolyl Blue Tetrazolium Bromide (MTT; Sigma-Aldrich, Vienna, Austria) and incubated for 1 h at 37 °C. The cells were then lysed by adding 100 *μ*l DMSO. Absorbance was recorded by a BioTek Synergy HT plate reader (BioTek Instruments, Winooski, VT, USA) at 570 nm. All experiments were run in triplicates.

### Cell cycle analysis, cell proliferation and apoptosis

Small cell lung cancer cells (0.4 × 10^6^ per ml) were seeded in 25 cm^2^ tissue culture flasks and then incubated with RAD001, erlotinib or with a combination of both at concentrations as indicated. After 48 h, cells were harvested and propidium iodide staining was performed as reported by Sieghart *et al* (2007). Cell cycle analysis (including sub-G1 peak for apoptosis) was performed using a FACSCalibur flow cytometer (Becton Dickinson, Heidelberg, Germany) and cell cycle distribution was calculated using ModFit LT software (Verity Software House, Topsham, ME, USA). To further analyse apoptosis, we performed DAPI staining according to the study group of Dornetshuber *et al* (2007). Cell proliferation was measured using the 3H-thymidine incorporation assay (Dornetshuber *et al*, 2007) 24 h after treatment of SCLC cells with erlotinib, RAD001 or a combination of both at indicated concentrations.

### Quantification of autophagy by an analysis of acidic vesicular organelle-positive cells

The effect of the investigated compounds alone and in combination on autophagy was assessed in both SCLC models by quantification of acidic vesicular organelles. In brief, cells were seeded at 3 × 10^5^ per well in six-well plates and treated with erlotinib, RAD001 and their combination at concentrations as indicated. After 48 h, cells were trypsinised, washed in PBS and stained with acridine orange (1 *μ*g ml^–1^ in serum- and phenol red-free medium; Sigma-Aldrich) for 15 min at 37 °C. The cells were analysed through flow cytometry for green (510–530 nm) and red (>650 nm) fluorescence emission from 2 × 10^4^ cells illuminated with blue (488 nm) excitation light, and were measured with FACSCalibur (Becton Dickinson) using CellQuest software. Cells positive for red fluorescence were scored positive for acidic vesicular organelles.

### EGFR sequencing in SCLC cells

Genomic DNA used as template for sequencing EGFR exons 18–21 was extracted from the GLC-4 and VL-68 cell lines as described previously (Lynch *et al*, 2004). In brief, polymerase chain reaction (PCR) fragments were sequenced and analysed in both sense and anti-sense directions for the presence of heterozygous mutations. The primer sequences for exon 18 were 5′-CTGAGGTGACCCTTGTCTCTG-3′ (forward) and 5′-CCAAACACTCAGTGAAACAAAGAG-3′ (reverse); for exon 19 5′-TGCCAGTTAACGTCTTCCTT-3′ (forward) and 5′-CAGGGTCTAGAGCAGAGCAG-3′ (reverse); for exon 20 5′-CATTCATGCGTCTTCACCTG-3′ (forward) and 5′-TTATCTCCCCTCCCCGTATC-3′ (reverse); and for exon 21 5′-CTTCCCATGATGATCTGTCC-3′ (forward) and 5′-TTATCTCCCCTCCCCGTATC-3′ (reverse). Mutations were identified by visual analysis of the sequence chromatograms using SeqScape (Applied Biosystems, Foster City, CA, USA).

### Western blot

Cells (2 × 10^6^ cells per well) in the logarithmic phase of growth were plated in 2 ml complete medium in six-well plates and allowed to attach overnight. Cells were treated with RAD001, erlotinib or with a combination of both at concentrations as indicated. They were then incubated for 72 h at 37 °C in a humidified atmosphere containing 5% CO_2_. After harvesting of the probes they were blotted according to standard procedures and incubated with monoclonal antibodies binding to EGFR (Santa Cruz Biotechnology, Santa Cruz, CA, USA, 1 : 1000), p-AKT (1 : 5000), p-mTOR (1 : 3000), p-p70s6K (1 : 3000), p-ERK (all from Cell Signaling, 1 : 5000) or Actin (Sigma-Aldrich; 1 : 40 000), respectively. Reactive bands were detected by chemiluminescence (CSPD substrate; Tropix Inc., Bedford, MA, USA). Equal protein loading in each lane was documented by detecting Actin protein expression.

### Statistical analysis

Association of different protein immunoreactivity among each other and with clinicopathological parameters was investigated using the *χ*^2^ test. Disease-free and overall survival was assessed using the Kaplan–Meier method. Cell culture data are presented as mean±s.d. Differences among treatment groups were calculated using one-way ANOVA, and Bonferroni's test was used for *post hoc* comparisons. For all tests, a two-tailed *P*-value of ⩽0.05 was considered significant.

## Results

### EGFR pathway is expressed in SCLC

**Figure 1:**
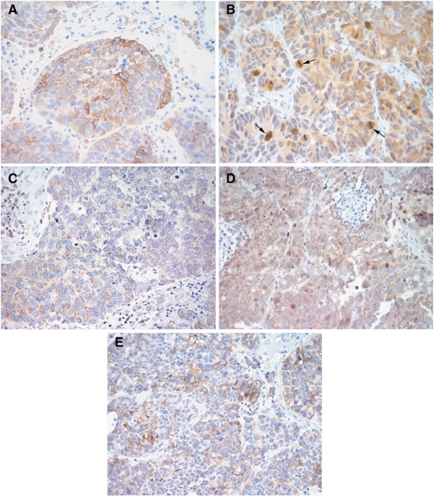
Immunostaining of EGFR and mTOR pathways in SCLC. Immunohistochemical staining of SCLC for (**A**) p-mTOR, (**B**) p-p70s6K (strongly stained mitoses are marked by arrows), (**C**) p-AKT, (**D**) p-ERK and (**E**) EGFR (all magnification × 400).

Overall, EGFR, p-AKT and p-ERK expression was detected in 37, 24 and 13% of tumour specimens (Figure 1 and Table 1). P-AKT and p-ERK showed cytoplasmatic staining pattern, whereas EGFR was expressed cytoplasmatic and membranous. The expression of EGFR and p-ERK as well as of p-AKT and p-ERK was positively associated (*P*=0.02 and *P*=0.0001), whereas EGFR and p-AKT expression was not (*P*>0.05, Table 2). Patients receiving preoperative chemotherapy were less likely to present with EGFR-positive tumours than patients without preoperative chemotherapy (14 *vs* 40%, *P*=0.038), but no influence of preoperative chemotherapy on p-AKT or p-ERK tumour expression was found (both *P*>0.05, Table 2). The expression of EGFR, p-AKT and p-ERK was not associated with tumour stage, disease-free or overall survival on univariate analysis (all *P*>0.05, Table 2).

### mTOR pathway is active in SCLC

Phospho-mTOR and p-p70s6K immunoreactivities were detected in 55 and 91% tumour specimens of all SCLC patients (Figure 1 and Table 1). Phospho-mTOR was expressed in the cytoplasma and p-p70s6K was detected in the cytoplasma and/or in the nucleus. Mitoses showed a remarkable cytoplasmatic p-p70s6K staining pattern (see arrows in Figure 1B). There was a significant association of p-mTOR and p-p70s6K (*P*=0.001, Table 2). Higher tumour stages were associated with lower p-mTOR expression (*P*=0.048), but no association between p-mTOR expression and nodal stage was found (*P*>0.05, Table 2). Higher tumour and nodal stages presented with lower p-p70s6K tumour expression (*P*=0.02 and *P*=0.001, Table 2). Preoperative chemotherapy had no influence on p-mTOR and p-p70s6K tumour expression. Phospho-mTOR and p-p70s6K tumour expression had no influence on disease-free and overall survival in univariate analysis (*P*>0.05, Table 2).

### EGFR and mTOR pathways are coexpressed in SCLC

In all, 28% of all patients showed coexpression of both pathways in terms of EGFR, p-mTOR and p-p70s6K positivity in SCLC tumour specimens. There was no significant difference in tumour or nodal stage distribution and administration of preoperative chemotherapy compared with patients without pathway coexpression (all *P*>0.05). A positive association between EGFR and p-mTOR expression (*P*=0.0001), but not between EGFR and p-p70s6K expression (*P*>0.05, Table 2), was found. There was no association between p-ERK, p-mTOR and p-p70s6K as well as p-AKT, p-mTOR and p-p70s6K tumour expression (all *P*>0.05, Table 2).

### Synergistic effects of erlotinib and RAD001 combination therapy on SCLC cells

**Figure 2:**
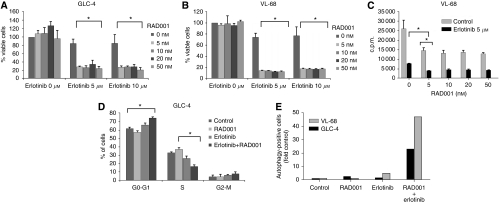
Effects on cell growth after treatment of SCLC cells with erlotinib, RAD001 and a combination of both. GLC-4 (**A**) and VL-68 (**B**) cells were treated with increasing doses of RAD001, erlotinib or a combination of both for 72 h and analysed for cell viability using the MTT assay. Data are given as mean percentage of viable cells±s.d. ^*^Statistical significance (*P*<0.05). (**C**) VL-68 cells were treated with erlotinib 5 *μ*M or increasing doses of RAD001 as indicated or a combination of both for 24 h, and thereafter were analysed using the ^3^H-thymidine assay. Data are given as mean counts per min±s.d. ^*^Statistical significance (*P*<0.05). (**D**) GLC-4 cells were treated with 5 *μ*M erlotinib, 10 nM RAD001 or a combination of both for 24 h and analysed by FACS after propidium iodide staining. Data are given as mean percentage of cells±s.d. ^*^Statistical significance (*P*<0.05). (**E**) VL-68 and GLC-4 cells were treated with 5 *μ*M erlotinib and 100 nM RAD001 or a combination of both for 48 h and analysed for acidic vesicular organelles. Data are given as x-fold autophagy-positive cells – one of three representative experiments is shown.

Given a potential target population of 28% patients coexpressing EGFR and mTOR pathways, we then evaluated the efficacy of RAD001 and erlotinib in SCLC cell lines (Figure 2A and B). Small cell lung cancer cells were treated with increasing doses of RAD001 (5, 10, 20 and 50 nM) and erlotinib (5 and 10 *μ*M) for 72 h.

RAD001 at doses of 5–50 nM had no significant anti-tumour effect. However, 5 *μ*M of erlotinib achieved a mild reduction of viable GLC-4 (16%) and VL-68 cells (26%) (see Figure 2A and B, *P*<0.001 compared with control) and this effect could not be increased using 10 *μ*M of erlotinib. We did not use higher doses of erlotinib, as 5 *μ*M erlotinib correspond to plasma concentrations in humans that can be achieved after oral dosing with 150 mg erlotinib per day (Hidalgo *et al*, 2001).

Combination treatment of VL-68 and GLC-4 cells (Figure 2A and B) revealed a strong, significant reduction of cell viability by up to 86% (s.d.±0.74%) and 72% (s.d.±2%), respectively, compared with the untreated control and the respective erlotinib or RAD001 monotherapy (all *P*<0.0001).

For further mechanistic insights, we analysed respective drug effects on cell DNA synthesis (^3^H-thymidine incorporation assay), cell cycle phases (PI staining), autophagy (acidic vesicular organelles) and apoptosis (DAPI staining and sub-G1 peak in cell cycle analysis). Treatment of VL-68 cells with 5 *μ*M erlotinib for 24 h revealed a strong reduction of DNA synthesis by 74% compared with control (Figure 2C). Addition of RAD001 at all doses further decreased DNA synthesis of erlotinib to 14% of untreated control (*P*<0.003, compared with erlotinib or RAD001 monotherapy, respectively), thus showing a cooperative inhibitory effect of this drug combination on SCLC cell proliferation. Similar results were observed for the GLC-4 cell line (data not shown) and confirmed by cell cycle analysis. Cell cycle analysis showed a significant G0/G1 arrest with a corresponding significant reduction of S phase of tumour cells upon combination treatment compared with respective controls (see Figure 2D). Although apoptosis was no major contributor to the observed anti-tumour effects (unremarkable DAPI staining, no evidence for a sub-G1 peak in FACS analysis, data not shown), there was a significant effect of combinatorial therapy on autophagy. Treatment of VL-68 and GLC-4 cell lines with erlotinib and RAD001 led to a 22- and 47-fold induction of autophagy compared with control whereas respective monotherapies revealed only a little effect (Figure 2E).

### Regulation of EGFR and mTOR pathways by erlotinib and RAD001 monotherapy and upon combination

**Figure 3:**
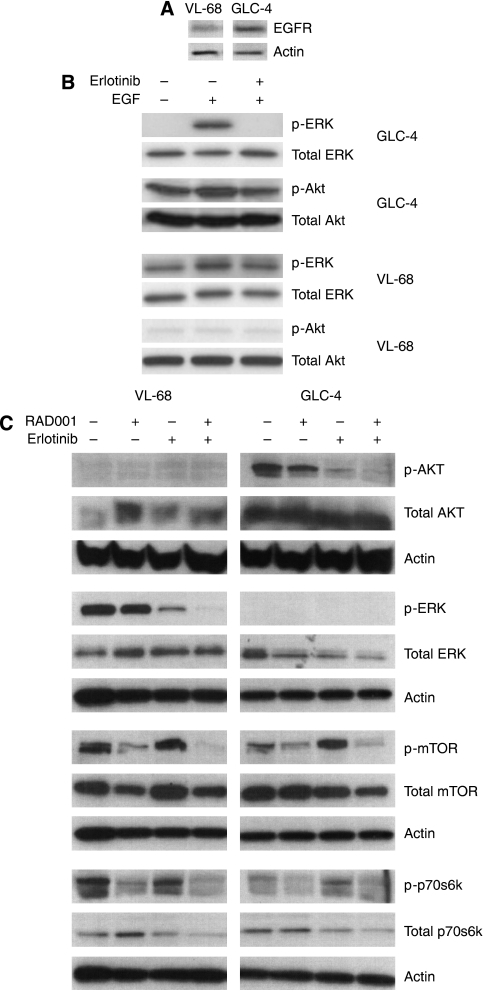
Effects on EGFR and mTOR pathways after treatment of SCLC cells with erlotinib, RAD001 and a combination of both. (**A**) GLC-4 and VL-68 cells do express EGFR. (**B**) GCL-4 and VL-68 cells were treated with 5 *μ*M erlotinib±EGF (100 ng ml^–1^) for 10 min and blotted for p-ERK, p-AKT and respective total proteins. (**C**) VL-68 and GCL-4 cells were treated with 5 *μ*M erlotinib, 5 nM RAD001 or a combination of both for 24 h, and then immunoblotted for total and phospho-protein expression of AKT, ERK, mTOR and p70s6K.

Given the strong synergistic antitumour effect of erlotinib and RAD001 combination therapy compared with the respective monotherapy, we evaluated the mechanism of action of this combination by monitoring therapy-associated changes in both pathways (Figure 3A–C); both cell lines showed a weak EGFR expression on the protein level (Figure 3A). No mutations were found in EGFR gene exon 18–21. We next tested whether the EGFR was functional in both cell lines. Thus, SCLC cells were serum starved for 24 h, followed by specific EGFR stimulation using 100 ng ml^–1^ EGF in the presence or absence of erlotinib (Figure 3B). Stimulation of SCLC cells caused a significant induction of p-ERK in both cell lines. Erlotinib blocked EGF-derived p-ERK induction in the GLC-4 cell line and reduced p-ERK induction to baseline levels in the VL-68 cell line (Figure 3B). The same was true for p-AKT in the GLC-4 cell line, whereas p-AKT was not inducible in the VL-68 cell line, a cell line lacking basal p-AKT expression. These data confirmed that the EGFR was functional in both cell lines and activated downstream targets upon activation with its ligand EGF.

Finally, we evaluated the effects of both drugs alone and upon combination on both cell lines: erlotinib monotherapy of GLC-4 cells with 5 *μ*M resulted in a clear downregulation of p-AKT, and significantly activated the mTOR pathway in terms of p-mTOR upregulation (Figure 3C), whereas erlotinib monotherapy of the p-AKT-negative VL-68 cell line with 5 *μ*M caused a significant downregulation of p-ERK levels, and – similarly to the GCL-4 cell line – significantly activated the mTOR pathway in terms of p-mTOR upregulation (Figure 3C). RAD001 treatment of GLC-4 and VL-68 cells with 5 nM resulted in the downregulation of p-mTOR and p-p70s6K. In addition, RAD001 influenced the EGFR pathway: there was a slight downregulation of p-ERK and p-AKT in the VL-68 and GLC-4 cell line, respectively. The combination of 5 *μ*M erlotinib and 5 nM RAD001 in GCl-4 cells caused a synergistic downregulation of p-AKT compared with erlotinib and RAD001 monotherapy. In the VL-68 cell line, the combination therapy synergised in terms of p-ERK downregulation compared with erlotinib monotherapy. In both cell lines, the mTOR pathway activation caused by erlotinib monotherapy was inhibited upon combination with RAD001.

## Discussion

Preclinical studies suggested synergistic effects upon combined EGFR and mTOR pathway inhibition in non-SCLC and breast (Buck *et al*, 2006), squamous cell carcinoma (Jimeno *et al*, 2007), glioblastoma (Wang *et al*, 2006), colon (Bianco *et al*, 2008), pancreatic cancer (Azzariti *et al*, 2008) and biliary tract cancer (Herberger *et al*, 2009). This is the first study testing the combination of EGFR targeting therapy with mTOR inhibitors for SCLC treatment.

First, we assessed single EGFR and mTOR pathway expression in 107 SCLC tissues. Surprisingly, EGFR receptor was expressed in 37% (see Table 2), which is more frequently than reported previously (Kaseda *et al*, 1989). In contrast, we found lower p-AKT and p-ERK expression when compared with other investigators (Blackhall *et al*, 2003). Moreover, we showed that expression of EGFR significantly correlated with its downstream target p-ERK. These results confirm *in vivo* the signalling information reported in various *in vitro* studies. Interestingly, patients receiving neoadjuvant chemotherapy with cisplatin and etoposid showed significantly lower EGFR expression than patients receiving no chemotherapy. Whether this reflects a true loss of EGFR receptor or a selection of EGFR-negative tumour cells remains to be investigated. However, reduced EGFR expression after chemotherapy could be, at least in part, responsible for the recent failure of a phase II clinical trial (Moore *et al*, 2006), testing gefitinib in chemotherapy pretreated SCLC patients.

The mTOR pathway was active in a significant proportion of patients in terms of p-mTOR (55%) and p-p70s6K (84%) expression (see Table 2). Similar to the association of EGFR and p-ERK, p-mTOR also showed its well-demonstrated association with p-p70s6K *in vivo*. Interestingly, mTOR pathway activation was stronger in earlier stages of disease. This finding could be important for future trial designs testing mTOR inhibitors in SCLC: a recently reported phase II clinical trial with the mTOR inhibitor everolimus (Owonikoko *et al*, 2008) in SCLC patients – including predominantly patients at an advanced stage of disease – failed to show significant clinical activity. In summary, EGFR and mTOR pathways were active in a significant proportion of patients with SCLC. Furthermore, 28% of SCLC patients showed coexpression of both pathways and may therefore represent the potential target population for combined anti-EGFR and mTOR targeting therapy.

Thus, we evaluated the efficacy of single- and dual-pathway inhibition in SCLC cell lines and investigated the mechanism of action of this combination at the molecular level. We found a synergistic antitumour effect upon combination of both drugs. The underlying mechanisms of the observed effect are complex and involve: (1) significant reduction of DNA synthesis (see Figure 2C), (2) a G0/G1 arrest with consecutive reduction of S phase (see Figure 2D), and (3) induction of autophagy (see Figure 2E), a mechanism that has been recently reported for mTOR inhibitors (Kim *et al*, 2006; Iwamaru *et al*, 2007; Crazzolara *et al*, 2009). In combination, these effects significantly contributed to the observed antitumour effect, whereas apoptosis did not seem to play a major role (unremarkable DAPI staining, no evidence for a sub-G1 peak in FACS analysis).

It is noteworthy that there was only a mild effect of erlotinib at a physiological dose in both cell lines (see Figure 2A and B) despite strong downregulation of p-AKT in GLC-4 cells and p-ERK in VL-68 cells (see Figure 3C). This might be explained by the absence of an activating EGFR mutation, which is a striking predictor for sensitivity of tumour cells to EGFR targeting therapy (Lynch *et al*, 2004; Ono and Kuwano, 2006). Compatibly, the only reported case showing clinical response was achieved in a patient carrying one of these activating mutations (Okamoto *et al*, 2006). Furthermore, the induction of mTOR signalling by erlotinib observed in this study may have contributed to the blunting of antitumour activity.

Similar to erlotinib, the efficacy of RAD001 monotherapy was not dose dependent and was lacking in both cell lines, despite target regulation in terms of p-mTOR and p-p70s6K downregulation. In contrast to previous reports (Buck *et al*, 2006; O’Reilly *et al*, 2006; Sieghart *et al*, 2007), there was no increase in AKT phosphorylation, which could have caused RAD001 resistance, leaving the mechanism of resistance unclear. Taken together, single EGFR or mTOR pathway inhibition seems ineffective for SCLC treatment *in vitro*, which may explain recent failures of mTOR and EGFR monotherapy in clinical trials of SCLC (Moore *et al*, 2006; Owonikoko *et al*, 2008).

Crucially, dual inhibition of EGFR and mTOR pathways by erlotinib and RAD001 combination therapy showed a synergistic and highly significant antitumour effect that could be explained on the molecular level by synergistic regulation of both pathways (see Figure 3C).

In summary, we found that the EGFR and mTOR pathways are active and coexpressed in a significant proportion of SCLC patients. A combination of erlotinib and RAD001 showed a synergistic antitumour effect, which was reflected on the molecular level, whereas respective monotherapies failed to prove significant antitumour efficacy. Therefore, this study provides a preclinical rationale to test dual inhibition of EGFR and mTOR pathways in SCLC in a prospective clinical trial.

## Figures and Tables

**Table 1:** EGFR and mTOR pathway immunostaining in 107 SCLC tissue specimens

**Immunohistochemical staining**	**Negative**	**Weak**	**Strong**
EGFR	67 (63%)	24 (22%)	16 (15%)
p-ERK	93 (87%)	8 (7%)	6 (6%)
p-AKT	81 (76%)	20 (19%)	6 (5%)
p-mTOR	48 (45%)	41 (38%)	18 (17%)
p-p70s6K	10 (9%)	40 (38%)	57 (53%)

Abbreviations: EGFR=epidermal growth factor receptor; mTOR=mammalian target of rapamycin; p-AKT=phosphorylated AKT; p-ERK=phosphorylated extracellular signal-regulated kinase; SCLC=small cell lung cancer.

**Table 2:** Association between EGFR and mTOR pathways and clinical–pathological parameters (*n*=107)

	**EGFR**	**p-ERK**	**p-AKT**	**p-mTOR**	**p-p70s6K**	**ChemoTx**	**pT-stage**	**pN-stage**	**DFS**	**OS**
EGFR		*P*=0.02	NS	*P*=0.0001	NS	*P*=0.038	NS	NS	NS	NS
p-ERK	*P*=0.02		*P*=0.0001	NS	NS	NS	NS	NS	NS	NS
p-AKT	NS	*P*=0.0001		NS	NS	NS	NS	NS	NS	NS
p-mTOR	*P*=0.0001	NS	NS		*P*=0.001	NS	*P*=0.048	NS	NS	NS
p-p70s6K	NS	NS	NS	*P*=0.001		NS	*P*=0.02	*P*=0.001	NS	NS

Abbreviations: DFS=disease-free survival; EGFR=epidermal growth factor receptor; mTOR=mammalian target of rapamycin; NS=not significant; OS=overall survival; p-AKT=phosphorylated AKT; p-ERK=phosphorylated extracellular signal-regulated kinase.
